# External validation of prognostic models for recovery in patients with neck pain

**DOI:** 10.1016/j.bjpt.2021.06.001

**Published:** 2021-07-01

**Authors:** Roel W. Wingbermühle, Martijn W. Heymans, Emiel van Trijffel, Alessandro Chiarotto, Bart Koes, Arianne P. Verhagen

**Affiliations:** a SOMT University of Physiotherapy, Amersfoort, the Netherlands; b Department of General Practice, Erasmus MC, Rotterdam, the Netherlands; c Department of Epidemiology and Biostatistics, VU University Medical Center, Amsterdam, the Netherlands; d Experimental Anatomy Research Department, Department of Physical Therapy, Human physiology and Anatomy, Faculty of Physical Education and Physical Therapy, Vrije Universiteit Brussels, Brussels, Belgium; e Department of Sports Science and Clinical Biomechanics, Faculty of Health Sciences, University of Southern Denmark, Odense, Denmark; f University of Technology Sydney, Sydney, Australia

**Keywords:** External validation, Neck pain, Prediction model, Prognosis, Prognostic model, Recovery

## Abstract

**Background:**

Neck pain is one of the leading causes of disability in most countries and it is likely to increase further. Numerous prognostic models for people with neck pain have been developed, few have been validated. In a recent systematic review, external validation of three promising models was advised before they can be used in clinical practice.

**Objective:**

The purpose of this study was to externally validate three promising models that predict neck pain recovery in primary care.

**Methods:**

This validation cohort consisted of 1311 patients with neck pain of any duration who were prospectively recruited and treated by 345 manual therapists in the Netherlands. Outcome measures were disability (Neck Disability Index) and recovery (Global Perceived Effect Scale) post-treatment and at 1-year follow-up. The assessed models were an Australian Whiplash-Associated Disorders (WAD) model (Amodel), a multicenter WAD model (Mmodel), and a Dutch non-specific neck pain model (Dmodel). Models’ discrimination and calibration were evaluated.

**Results:**

The Dmodel and Amodel discriminative performance (AUC < 0.70) and calibration measures (slope largely different from 1) were poor. The Mmodel could not be evaluated since several variables nor their proxies were available.

**Conclusions:**

External validation of promising prognostic models for neck pain recovery was not successful and their clinical use cannot be recommended. We advise clinicians to underpin their current clinical reasoning process with evidence-based individual prognostic factors for recovery. Further research on finding new prognostic factors and developing and validating models with up-to-date methodology is needed for recovery in patients with neck pain in primary care.

## Introduction

Neck pain is common and one of the leading causes of disability in most countries.^1^^,^^2^ From 2005 to 2015, prevalence of chronic neck pain has increased globally by 21.1% and is likely to increase further.^1^^,^^2^ Recovery from neck pain-related disability mainly takes place in the first few weeks without further subsequent improvement.^3^ Acute neck pain prognosis may be even worse than currently recognized which underlines the importance of neck pain prognosis at intake in primary care.^3^

Short-term beneficial effects and cost-effectiveness of non-invasive primary care treatment have been reported but long-term effects are still limited.^4^, ^5^, ^6^, ^7^ Prognostic models are obtained by multivariable regression and aim to improve the quality of care for *individual* patients by estimating the probability of a future health outcome or condition being present by combining *patient specific values* of multiple predictors.^8^ Accurate prognostic models can be useful for clinicians to support clinical decisions and for research to risk-stratify participants for clinical trials.^8^, ^9^, ^10^ Compared to derivation studies, models usually perform less well in external validation studies and it is recommended first to test models’ generalizability and transportability to evaluate whether their predictive performance remains accurate before broad clinical use can be advised.^11^, ^12^, ^13^

Numerous prognostic models for people with neck pain have been developed, however, few have been validated.^14^, ^15^, ^16^ In a recent systematic review, three promising models that predict recovery of people with neck pain in primary care were identified.^17^ However, their broad clinical use could not be recommended and further external validation was advised.^17^ Therefore, the research question of this study was: can these three models be externally validated in a cohort of people with nonspecific neck pain treated with manual therapy in Dutch primary care?

## Methods

This external validation study including its statistical analysis was performed according to an a priori constructed and approved study protocol complying with internal university procedures. The included models were: 1) the Australian two-way model (Amodel)^18^ predicting full recovery and ongoing moderate to severe disability, measured with the Neck Disability Index (NDI) in patients with Whiplash-Associated Disorders (WAD); 2) the multicenter model (Mmodel)^19^ also predicting disability measured with the NDI in patients with WAD, and 3) the Dutch model (Dmodel)^20^ predicting recovery measured with a Global Perceived Effect Scale (GPES) in patients with non-specific neck pain. Models’ characteristics are presented in Table 1. The findings of this study were reported according to the Transparent Reporting of a multivariable prediction model for Individual Prognosis Or Diagnosis (TRIPOD) recommendations.^21^

**Table 1:** Models’ characteristics.

	First author and year	Setting	Condition, treatment and number of participants	Participants characteristics	Outcomes, follow up	Models with intercept, predictors and their weights
**Amodel**	Ritchie et al. 2013	Australian hospital accident and emergency departments, primary care practices, and recruitment from advertisement	WAD-acute, grade 1,2 or 3; usual care not withheld from; *n* = 336	Mean age 36.4 years. Mean VAS pain: 4,2	**Full recovery:** Function at 12 months NDI score multiplied by two and cutoff ≤ 10%	−1.667; 1.856 NDI initial ≤ 32, 0.717 Age ≤ 35
					**Ongoing disability:** Function at 12 months NDI score multiplied by two and cutoff ≥ 30%	−2.859; 2.013 NDI initial ≥ 40; 0.811Age ≥ 35, 0.796 Hyper arousal subscale (PDS) ≥ 6
**Mmodel**	Sterling et al. 2005	Australian hospital accident and emergency departments, primary care practices, and recruitment from advertisement	WAD acute, grade 2 or 3; Free to pursue any treatment; *n* = 80	Mean age 36.2 (SD12.6) years. 70% female Mean NDI 34.15 (SD 2.37)	**Persistent neck complaints:** Function at 6 months, NDI score	11.74; 0.387 Initial NDI score; 0.387 Age, −0.178 ROM Left rotation; 0.505 CPT; 0.338 IES; −0.0147 QI
**Dmodel**	Schellingerhout et al. 2010	Dutch primary care settings	Neck pain nonspecific; different therapy in RCT (usual care GP, PT, MT, graded activity); *n* = 468	Mean age 45.4 (SD 11.8) years. 61% female NDI 14.5/50 (SD 6.7)	**Recovery**: GPRS at 6 months, dichotomized into recovered or much improved and persistent complaints	−1.704; 0.029 Age, −0.042 pain intensity, 0.198 headache, −0.564 radiation of pain to elbow/shoulder, 0.515 previous neck complaints, 0.234 cause of complaints, 0.829 low back pain, 0.372 employment status, 0.005 EuroQoL, 0.116 accompanying headache * pain intensity, −0.376 accompanying headache * previous neck complaints, 0.392 accompanying headache * radiation of pain, −0.815 accompanying headache * employment status

Abbreviations: WAD= Whiplash Associated Disorder; GP=General Practitioner; PT=Physical Therapy; MT=Manual Therapy; NPRS=Numeric Pain Rating Scale; VAS=Visual Analogue Scale; NDI=Neck Disability Index; GPRS=Global Perceived Recovery scale; EuroQoL=Quality of Life; ROM=Range Of Motion; IES=Impact of Events Scale; QI=Quotient of Intergrals in blood flow; CPT=Cold Pain Threshold. * indicates interaction terms in the regression models.

### ANIMO validation cohort

For validation, existing data from the ‘Amersfoorts Nekonderzoek of the Master manuele therapie Opleiding’ (ANIMO) study was used. Ethics approval was obtained from Erasmus Medical centre, Rotterdam, the Netherlands (MEC-2007-359). The dataset used and analyzed during the current study are available upon reasonable request. ANIMO is a prospective cohort study that aimed to describe usual care manual therapy for patients with neck pain in the Netherlands and explored outcomes and adverse events of treatment. Patients between 18 and 80 years with neck pain consulting a directly accessible manual therapist were recruited from October 2007 until March 2008. Participants with signed informed consent and treatment indication who submitted baseline data were eligible for participation (*n* = 1193). Received treatment consisted of usual care manual therapy and may have included specific joint mobilizations, high velocity thrust techniques, myofascial techniques, giving advice, or specific exercises. Further study characteristics are described in detail elsewhere.^22^

### Measurement procedure

Participants completed socio-demographic characteristics and questionnaires at baseline, immediately post-treatment, and at 12 months. Manual therapist where blinded from information gathered by patients’ questionnaires. At baseline, patients’ age, sex, marital status, employment, neck pain duration, neck pain localization, earlier episodes, associated symptoms, current medication, current smoking, current sport, imaging results, additional diagnostics, medical diagnosis, and comorbidities were recorded. Disability was measured using the Dutch versions of the NDI (scale 0–50)^23^^,^^24^ and the Neck Bournemouth Questionnaire (NBQ, scale 0–70)^25^; pain intensity was measured with a 10-point Numeric Rating Scale (NRS, scale 1–10), and pain-related fear was measured with the Dutch version of the Fear Avoidance Beliefs Questionnaire (FABQ-DV, scale 0–96).^26^ Outcomes were measured post-treatment at discharge (mean treatment duration 37.9 days, mean number of 4.3 sessions) and at 12 months follow-up, using the NDI and a GPES (7-point Likert scale).

### Validation procedure

Based on models’ predictors available in ANIMO, the Amodel(s) and Dmodel were suitable for validation.^20^^,^^27^ The Mmodel was considered not suitable due to four variables not collected in ANIMO (i.e. cold pain threshold, impact of events scale, quotient of a sympathetic vasoconstrictor response; left rotation) with lack of appropriate proxy measures.^28^ As the Amodel(s) were developed for people with WAD and ANIMO also contained patients with non-traumatic neck pain, we created a subset of patients with self-reported trauma in ANIMO. We used the NBQ anxious subscale with comparable cutoff value as proxy for the hyperarousal subscale of the Posttraumatic stress Diagnostic Scale (PDS) because the PDS was not available in ANIMO. For the Dmodel, we removed the quality of life variable (EuroQoL, beta value 0.005) because this was not available in ANIMO. We used the same outcome cut-off values as the original studies.

We examined baseline demographics, models’ predictors, and outcome distribution between the models’ development studies and ANIMO as means with standard deviations or frequencies or percentages to compare case-mix between studies.

#### Handling of missing values

The ANIMO data contained missing values and we planned to perform several missing value analyses to decide on multiple imputation for main analyses and complete cases for sensitivity analysis. ^29^^32^-

### Statistical analysis

#### Statistical validation of models’ performance

We compared observed outcomes to those predicted by the models and analyzed the full original models in ANIMO and based models’ performance on discrimination and calibration measures.^10^^,^^13^^,^^33^ The Amodel was analyzed in both the ANIMO trauma subset as well as the whole dataset. We calculated model's linear predictor and individual probability (p (*y* = 1) =1/ (1 + *e*^−linear predictor^)) for all participants immediately post-treatment and at 1 year follow-up.^34^

#### Discriminative performance

Discriminative performance indicates whether a model is able to distinguish between patients with and without recovery. It is calculated as the concordance (c) statistic which is comparable to the area under curve (AUC) of the Receiver Operating Characteristic curve (ROC) for binary data.^13^^,^^35^ We a priori considered discriminative performance acceptable if AUC was ≥ 0.70.^36^

#### Calibration performance

Calibration performance refers to the agreement between a model's predicted risks and observed event rates.^37^ Preferably, this is reflected by calibration-in-the large, a calibration slope, and a calibration plot.^13^^,^^38^ The Hosmer-Lemeshow goodness of fit test is often performed in validation studies and if the test is not-significant, it should indicate that the model fits the data well.^36^ The models were re-estimated in ANIMO on al logit scale with the linear predictor as only predictor to calculate calibration-in-the large and the calibration slope.^10^^,^^13^^,^^30^ We evaluated calibration as percentage of deviation from the ideal calibration slope of 1 and the intercept of 0. Calibration plots’ probabilities were calculated to allow observation if all decile groups closely fit the ideal 45° line of identity.^10^^,^^13^ We performed statistical validation procedures using IBM SPSS 24.0 and R (version 3.4.3).

Finally, we checked the number of events in ANIMO for a minimum of 100, as advised for validation studies that predict binary outcomes.^39^^,^^40^

## Results

### Study characteristics

The baseline characteristics from the ANIMO study and from the original studies are presented in Table 2.

**Table 2:** The baseline characteristics of participants in the ANIMO validation cohort and the original studies.

	ANIMO Validation cohort (*n* = 1193)		ANIMO Trauma validation sub cohort^e^ (*n* = 143)		Amodels Derivation study b (*n* = 262)	Dmodel Derivation study (*n* = 468)
	*Value*^a^ n (%)	*Missing n (%)*	*Value*^a^ n (%)	*Missing n (%)*	*Value*^a^ n (%)	*Value*^a^*n* (%)
**Baseline characteristics**						
Sex Female Male	823 (69.4%) 363 (30.6%)	7 (0.6%)	102 (71.8%) 40 (28.2%)	1 (0.7%)		182 (39%)
Duration current episode c Acute Subacute Chronic	420 (39.2%) 138 (12.9%) 513 (47.9%)	122 (10.2%)	49 (35.5%) 11 (08.0%) 78 (56.5%)	5 (3.5%)	262 (100%)	58 (13%) 225 (48%) 160 (34%)
Marital status, yes	889 (77.2%)	41 (3.4%)	102 (72.9%)	3 (2.1%)		
Currently smoking, yes	300 (25.2%)	3 (0.3%)	30 (21.0%)	0 (0.0%)		
Current medication use, yes	560 (47.1%)	3 (0.3%)	74 (51.7%)	0 (0.0%)		
Current sports, yes	783 (65.9%)	4 (0.3%)	93 (65%)	0 (0.0%)		
Disability (NDI), mean ± SD	13.0 ± 6.5	98 (8.2%)	15.9 ± 7.9	13 (9.1%)	16.5 ± 8.7	14.5 ± 6.7
Fear avoidance, FABQ scale 0–96 FABQ work subscale 0–66 FABQ physical activity subscale 0–30	1053 26.6 ± 16.6 1129 13.4 ± 12.2 1103 13.2 ± 7.3	140 (11.7%) 64 (5.4%) 90 (7.5%)	30.6 ± 18.6 16.0 ± 14.0 14.6 ± 7.4	15 (10.5%) 8 (5.6%) 10 (7.0%)		
Expected recovery by patient, scale 1–5 Much better Better No change Worse Much worse	1190 517 (43.4%) 662 (55.6%) 10 (00.8%) 1 (00.1%) 0 (0.00%)	3 (0.3%)	143 57 (39.3%) 83 (58.0%) 3 (02.1%) 0 (0.00%) 0 (0.00%)	0		
**Dmodel for persistent neck complaints**^d^						
Age, yrs.	1170 44.7 ± 13.7	23 (1.9%)	41.9 ± 13.8	1 (0.7%)	37.1 ± 14.2	45.4 ± 11.8
Pain, 11-point Likert scale g	1189 3.3 ± 2.7	4 (0.3%)			4.2 ± 2.1	5.7 ± 2.1
Headache, yes	707 (59.2%)		101 (70.6%)			317 (68%)
Radiating arm pain, yes	536 (44.9%)		66 (46.2%)			296 (63%)
Previous neck pain episode, yes	755 (66.9%)	64 (5.4%)	80 (59.3%)	8 (5.6%)		301(64%)
Cause of complaints trauma, yes	143 (13.0%)*	97 (8.1%)				63 (14%)
Low back pain	538 (45,1%)		65 (45.5%)			96 (21%)
Employed, yes	897 (77.1%)	29 (2.4%)	112 (79.4%)	2 (1.4%)		334 (71%)
Euro QoL 100^h^						69.9 ± 17.3
**Amodel for full recovery**						
NDI ≤ 32	180 (16.4%)		74 (56.9%)			
Age ≤ 35 yrs.	306 (26.2%)		49 (34.5%)			
**Amodel for moderate/severe disability**						
NDI ≥ 40	796 (72.7%)		40 (30.8%)			
Age ≥ 35 yrs.	888 (75.9%)		98 (69.0%)			
PDS hyperarousal subscale (0–15) f	481 (40.6%)	8 (0.7%)	69 (48.3%)		4.8 ± 3.8	
**Outcome characteristics**^i^						
**Post-treatment**						
Global Perceived Effect, 7-point Likert scale 0–70 Completely recovered Much improved Slightly improved No change Slightly worse Much worse Worse than ever	568 129 (22.7%) 317 (55.8%) 97 (17.1%) 25 (4.4%) 0 (0.0%) 0 (0.0%) 0 (0.0%)	625 (52.4%)	65 13 (20.0%) 38 (58.5%) 11 (16.9%) 3 (4.6%) 0 (0.0%) 0 (0.0%) 0 (0.0%)	78 (54.5%)		
Disability, NDI scale 0–50	541 12.1 ± 11.0	652 (54.7%)	64 8.0 ± 6.3	79 (55.2%)		
**Long term outcome**						
Global Perceived Effect, 7-point Likert scale 0–70 Completely recovered Much improved Slightly improved No change Slightly worse Much worse Worse than ever	685 157 (22.9%) 264 (38.5%) 153 (22.3%) 88 (12.8%) 12 (1.8%) 8 (1.2%) 3 (0.4%)	508 (42.6%)	86 19 (22.1%) 34 (39.5%) 18 (20.9%) 12 (14.0%) 1 (1.2%) 2 (2.3%) 0 (0.0%)	57 (39.9%)		
Disability, NDI scale 0–50	541 6.0 ± 5.4	515 (43.2%)	87 8.3 ± 8.0	56 (39.2%)		
**Dmodel for persistent neck complaints (GPE)**						
Post-treatment persistent complaints complete/much improved	122 (21.5%) 446 (78.5%)		14 (21.5%)			
Long-term persistent complaints complete/much improved	264 (38.5%) 421 (61.5%)		33 (38.4%) 51 (61,6%)			(43%)
**Amodel for full recovery**						
Post-treatment persistent complaints NDI	294 (54.3%)		51 (78.5%)			
Long term persistent complaints NDI	389 (57.4%)		41 (47.1%)		120 (46%)	
**Amodel for moderate/severe disability**						
Post-treatment persistent complaints NDI	40 (7.4%)		9 (14.1%)			
Long term persistent complaints NDI	45 (6.6%)		13 (14.9%)		69 (26%)	

Values are numbers (percentages) unless stated otherwise.

NDI = Neck Disability Index; FABQ = Fear Avoidance Beliefs Questionnaire; NRS = Numeric Rating Scale, euro QOL = Quality of Life; GPE = Global Perceived Effect; SD = Standard Deviation.

a Data presented as responders n (%) or mean ± SD.

b Complete cases of acute whiplash (*n* = 336 eligible).

c acute < 1 months, subacute 1–3 months, chronic >3 months.

d Constant and predictor's weight as Beta value.

e As any self-reported trauma, according to patient and/or therapist.

f in ANIMO Neck Bournemouth Questionnaire (NBQ) subscale ≥ 4 (how anxious, tense, uptight, irritable, difficulty concentrating/relaxing, as proxy for hyperarousal subscale of the posttraumatic stress diagnostic scale (PDS).

g In Dmodel studies as NRS 11-point Likert scale 0–10; in Amodel studies as VAS-scale; in ANIMO as NRS 1-point Likert scale 1–10.

h not available in ANIMO.

i Dmodel: GPE dichotomized as not complete + much improved; Amodel-moderate/severe complaints: dichotomized as NDI ≥ 30%; Amodel-full recovery: dichotomized as NDI ≤ 10%.

### Amodels

The ANIMO subset consisted of people with any trauma and neck pain duration, whereas the original Amodel study included people with acute neck pain due to a motor vehicle crash only. People in ANIMO were recruited and treated in primary care with manual therapy and people in the original study were allowed to pursue any treatment and where recruited from general advertisement and emergency departments. On average, people in the original study were 4.8 years younger compared to the ANIMO trauma subset, had 17 NDI points higher disability (0–50 scale), and had 0.9 point more pain (0–10 scale).

### Dmodel

There were 8.1% less male participants in ANIMO compared to the Dmodel derivation study. Duration of current episode in the Dmodel derivation cohort resulted in 26% more patients categorized as acute and 13.5% more categorized as chronic compared to ANIMO. In ANIMO, average disability at inception was 1.5 NDI points lower and the average neck pain was 2.4 points less on an 11-point Likert scale. For the other variables, there were 8.8% less people with headache and 20.1% less with radiating arm pain. In ANIMO, 2.9% more people had a previous neck pain episode, 24.1% more had concomitant low back pain, and 6.1% more people were employed.

### Missing data

There were more than 5% missing data for several baseline variables and all outcome measures (Table 2). Little's Missing Completely at Random (MCAR) test was significant at the *p*<0.05 level so we assumed data were not MCAR. Significant differences in means existed for 24 of 91 variables and differences were small indicating Missing at Random (MAR). Explained variation of missingness varied from 11 to 100% and missing variables were to some extent associated with the other ANIMO variables. Therefore, we assumed data were MAR.

We applied multiple regression imputation for missing data using all possible predictors and outcomes, as computationally feasible.^29^^,^^31^^,^^41^ We used the Multivariate Imputation by Chained Equations (MICE) procedure and generated 20 imputed sets.^42^ Regression coefficient estimates and standard errors were pooled using Rubin's Rules and validation performance measures were estimated in each of the 20 completed datasets and then combined using the median.^30^^,^^43^ We used imputed data for main analyses and complete cases for sensitivity analysis.

### Models’ performance

The ANIMO smallest outcome groups contained 122, 247, and 40 events at post-treatment for GPE, NDI recovery, and NDI moderate/severe, respectively. At long-term, these numbers were 264, 289, and 45, respectively. These numbers revealed sufficient sample size for the Dmodel and Amodel recovery post-treatment and at long-term. The ANIMO trauma subset did not have a sufficient sample size as it contained 24 recovered people as measured by the NDI and 9 with moderate/severe outcome post-treatment, and 41 and 13 at long-term.

### Discriminative performance

Models’ performance measures are described in Table 3.

**Table 3:** Model's performance measures.

	Discrimination (AUC) a	Calibration Slope b	Calibration In-the-large (intercept) b
**Amodel for full recovery**			
Post-treatment^c^	0.53 (0.24, 0.80)	−0.35 (−0.57, −0.30)	0.46 (0.13, 0.75)
Long term outcome^c^	0.49 (0.26, 0.72)	−0.26 (−0.30, −0.10)	0.34 (−0.04, 0.82)
Long term outcome^d^	0.43 (0.40, 0.49)		
**Amodel for moderate/severe disability**			
Post-treatment *	0.54 (0.40, 0.69)	−0.06 (−0.12, 0.00)	−0.63 (−1.06, −0.08)
Long term outcome *	0.54 (0.38, 0.69)	−0.01 (−0.04, 0.06)	−1.13 (−1.76, −0.79)
Long term outcome **	0.43 (0.34, 0.52)		
**Dmodel for persistent neck complaints,**			
Post-treatment	0.53 (0.48, 0.58)	−0.06 (−0.15, −0.06)	−0.97 (−1.03, −0.79)
Long term outcome	0.54 (0.49, 0.58)	0.23 (0.14, 0.28)	−0.33 (−0.39, −0.31)

Data analyzed on pooled data.

a As logit with 95% low and 95% up.

b As median with 1st and 3rd inter quartile range.

c A-models tested in ANIMO trauma subset.

d A-models tested in full ANIMO set.

Discriminative performance (analyzed in the trauma subset) of the Amodel that predicts full recovery immediately post-treatment was 0.53 (95% CI: 0.24, 0.80) and was 0.49 (95% CI: 0.26, 0.72) for long-term outcome. Discriminative performance of the Amodel that predicts ongoing moderate to severe disability post-treatment was 0.54 (95% CI: 0.40, 0.69) post-treatment and 0.54 (95% CI: 0.38, 0.69) for long-term outcome. Discriminative performance of the Dmodel was 0.53 (95% CI: 0.48, 0.58) post-treatment and 0.54 (95% CI: 0.49, 0.58) at long-term outcome. These results indicate poor discriminative performance of both models.

Analysis of the Amodels in the whole ANIMO cohort at long-term follow-up revealed a discriminative performance for the model that predicts full recovery of 0.43 (95% CI: 0.40, 0.49) and for the model that predicts ongoing moderate to severe disability of 0.43 (95% CI: 0.34, 0.52), also displaying poor discriminative performance.

### Calibration performance

Performance of calibration-in-the-large for the Amodel that predicts full recovery post-treatment was 0.46 (IQR: 0.13, 0.75) and 0.34 (IQR: −0.04, 0.82) for long-term outcome. The calibration slope was −0.35 (IQR: −0.57, −0.30) and −0.26 (IQR: −0.30, −0.10), respectively. For the Amodel that predicts ongoing moderate/severe disability post-treatment, calibration-in-the-large was −0.63 (IQR: −1.06, −0.08) and −1.13 (IQR: −1.76, −0.79) for long-term outcome. The calibration slope was −0,06 (IQR: −0.12, 0.00) and −0.01 (IQR: −0.04, 0.06), respectively. The Hosmer-Lemeshow goodness of fit test was significant for both Amodels.

**Fig. 1:**
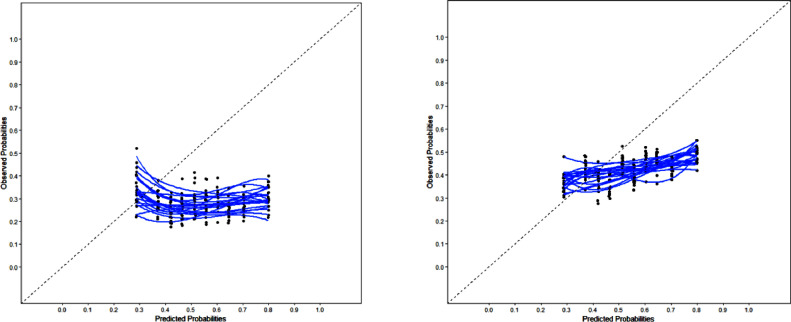
Calibration plots with 20 calibration lines (blue) of each imputed dataset. Predicted probabilities are plotted against actually observed outcomes in relation to the ideal 45° line of perfect prediction (dotted line) in ANIMO decile subgroups of predicted events. Ideally, all blue lines lay exactly on the dotted line. Dmodel long term outcome left figure, post treatment right figure.

Performance of calibration-in-the-large for the Dmodel was −0.97 (IQR: −1.03, −0.79) post-treatment and −0.33 (IQR: −0.39, −0.31) for long-term outcome. The calibration slope was −0.06 (IQR: −0.15, −0.06) and 0.23 (IQR: 0.14, 0.28), respectively. The Hosmer-Lemeshow goodness of fit test was significant for all D model outcomes. Dmodel calibration plots are shown in Fig. 1. These values deviate substantial from the intercept of 0 and the ideal calibration slope of 1 and show poor calibration of both models.

### Sensitivity analysis

Sensitivity analyses of discriminative performance in complete cases demonstrated lower c-statistics of 0.36 (95% CI: 0.31, 0.41) and 0.44 (95% CI: 0.39, 0.49) for the Amodel that predicts full recovery at post-treatment and long-term, respectively. For the Amodel that predicts ongoing moderate/severe disability, these values were 0.46 (95% CI: 0.36, 0.57) and 0.42 (95% CI: 0.34, 0.52), respectively. Dmodel's discriminative performance was 0.56 (95% CI: 0.50, 0.63) and 0.54 (95% CI: 0.50, 0.69), respectively. Also, complete case analyses displayed poor discriminative performance for all models.

## Discussion

External validation in a cohort of people with neck pain of a two-way WAD model (Amodel) that predicts disability measured by the NDI, and a non-specific neck pain model (Dmodel) that predicts recovery measured by the GPE, was not successful as their discriminative performance and calibration clearly did not meet expected thresholds. A third prognostic model could not be evaluated in this study because of variable discrepancy across data sets.

The Amodels’ discriminative performance was substantially below 0.70 for all time points. However, its discriminative and calibration performance could not be compared with the original studies because these measures were not described and our study is the first in presenting Amodels’ performance measures.^18^^,^^27^ The Amodel full recovery broad confidence intervals obtained in the trauma subset included AUC 0.70 values close to the upper bounds. These broad intervals could be explained by too few events, because the ANIMO trauma subset did not reach the minimum of 100 events in the smallest outcome group. Analysis in the whole ANIMO cohort, containing sufficient events, revealed small intervals but with 0.52 as the upper bound value.

The Dmodel's discriminative performance in the original study was 0.66 (95% CI: 0.61, 0.71) at internal validation and 0.65 (95% CI: 0.59, 0.71) at external validation. Our validation study revealed a lower 0.53 (95% CI: 0.48, 0.58) AUC post-treatment and 0.54 (95% CI: 0.49, 0.58) AUC for long-term predictions. A decrease in discriminative performance from derivation to validation is not unusual.^33^ Dmodel's performance at development was already below our cut-off 0.70 for AUC and a 0.12 decrease of an overfitted model in another population with different case-mix is not an unexpected finding. Additionally, there may be little distinction in AUC between our validation study and the development study, as the 95% CI are close together. In addition, calibration was poor for both Dmodel and Amodels. At external validation, predictions are often too extreme due to overfitting at the development phase.^44^ This results in low predictions being too low and high predictions being too high, as characterized by a calibration slope smaller than 1 and indicate that the original regression coefficients were too large.^13^^,^^45^^,^^46^ In addition, we believe case-mix differences could not have been responsible for models' poor performance as these differences were relatively small. Comparison of model performance to other studies in the field is hampered: prognostic prediction models in the musculoskeletal field typically do not reach their validation phase and methodological shortcomings are common. In fact, the few models that were evaluated for external validity usually did not present model performance by means of calibration and discrimination measures.^14^^,^^17^^,^^47^

### Strengths and limitations

Strength of our study is analysis in a large cohort by state-of-the-art calibration and discrimination measures. However, there are some limitations we would like to report. First, in ANIMO, multiple independent therapists at multiple sites were used and the broad CIs derived in the large ANIMO cohort could reflect this measurement variability. Second, the validation data set had substantial missing values, which is not unusual.^48^ We applied multiple imputation procedures and sensitivity analysis on complete cases that showed comparable values of the performance measures. Third, the EuroQol predictor for the Dmodel and the hyperarousal subscale predictor for the first Amodel were not available in ANIMO and may have influenced model performance. However, this impact is probably negligible considering the 0.005 bèta value for EuroQol. We believe that the NBQ anxious subscale predictor served sufficiently as proxy for the hyperarousal subscale, thereby, the other Amodel that did not contain this predictor performed very similar. Fourth, the predicted outcomes for the Dmodel at derivation and validation were measured at 6 months and 12 months, respectively. We believe that the impact of these different outcome times is limited as overall prognosis for neck pain and disability for 6 and 12 months appear to be similar.^49^

### Implications for practice and research

Based on our findings, the clinical use of these promising models can, at present, not be advocated. We feel this is a very important message for musculoskeletal clinicians considering the numerous models that predict outcomes in neck pain that are available for clinicians without this crucial step of subsequent external validation, which could potentially lead to undesired outcomes for patients when models are implemented too early in practice. We advise clinicians to underpin their clinical reasoning process at this moment with separate prognostic factors that can be used with more confidence, such as baseline pain intensity, baseline neck disability, age, and past history of musculoskeletal disorders.^50^

The low performance of the existing prognostic models indicate that important predictors may not have been included in the models’ derivation process and further search for valuable model predictors is needed.

## Conclusion

External validation of two promising prognostic models on neck pain recovery in primary care was not successful and their clinical use can, at present, not be advocated. Currently, no useful models are available for clinicians to predict outcomes in people with neck pain. New insights on potentially valuable prognostic factors are needed to strengthen models’ derivation and updating procedures.

## Conflict of Interest

The authors declare no conflicts of interest
